# Pericytes in Cerebrovascular Diseases: An Emerging Therapeutic Target

**DOI:** 10.3389/fncel.2019.00519

**Published:** 2019-11-22

**Authors:** Xiaojuan Su, Lingyi Huang, Yi Qu, Dongqiong Xiao, Dezhi Mu

**Affiliations:** ^1^Department of Paediatrics, West China Second University Hospital, Sichuan University, Chengdu, China; ^2^Key Laboratory of Birth Defects and Related Diseases of Women and Children, Sichuan University, Ministry of Education, Chengdu, China; ^3^West China College of Stomatology, Sichuan University, Chengdu, China

**Keywords:** pericytes, angiogenesis, basement membrane, blood brain barrier, cerebrovascular disease

## Abstract

Pericytes are functional components of the neurovascular unit (NVU) that are located around the blood vessels, and their roles in the regulation of cerebral health and diseases has been reported. Currently, the potential properties of pericytes as emerging therapeutic targets for cerebrovascular diseases have attracted considerable attention. Nonetheless, few reviews have comprehensively discussed pericytes and their roles in cerebrovascular diseases. Therefore, in this review, we not only summarized and described the basic characteristics of pericytes but also focused on clarifying the new understanding about the roles of pericytes in the pathogenesis of cerebrovascular diseases, including white matter injury (WMI), hypoxic–ischemic brain damage, depression, neovascular insufficiency disease, and Alzheimer’s disease (AD). Furthermore, we summarized the current therapeutic strategies targeting pericytes for cerebrovascular diseases. Collectively, this review is aimed at providing a comprehensive understanding of pericytes and new insights about the use of pericytes as novel therapeutic targets for cerebrovascular diseases.

## Introduction

Pericytes are among the major components of vascular composition. They are located along the capillary walls at intervals and possess numerous biological functions and characteristics. In particular, pericytes exhibit migratory, immune, phagocytic, and cytoprotective functions as well as stem cell potential and are involved in tissue repair (Attwell et al., 2016). In the CNS, pericytes participate in brain self-regulation, which is vital for blood vessel formation, maintenance of BBB integrity, regulation of cerebral blood flow, and coagulation (Santos et al., 2018). Additionally, pericytes are involved in the pathogenesis and development of various diseases. For instance, under ischemic conditions, pericytes constrict the capillaries in rigidity and hinder the flow of blood cells, which eventually prevents microcirculatory reperfusion even if the plaque has been removed in stroke disorders (Beazley-Long et al., 2018). Furthermore, studies have indicated that pericytes are associated with other neurovascular disorders, including WMI, cerebral hemorrhage, and hypoxic–ischemic brain damage (Hill et al., 2014). Therefore, after gaining a comprehensive understanding of the characteristics of pericytes and the in-depth studies on their relationship with diseases, scientists have regarded pericytes as good targets for the treatment of diseases. Although pericytes have been the subject matter of various studies, only few reviews have focused on discussing pericytes in cerebrovascular diseases, and data on the characteristics of pericytes and their use as therapeutic targets remain insufficient (Lange et al., 2013). Thus, we decided to conduct a review that firstly provided sufficient knowledge about this topic.

To provide a comprehensive understanding of pericytes, in this review, we summarized the biological characteristics of pericytes in detail, including their research history, distribution, isolation and culture methods, identification strategies, and biological functions. In addition, we elucidated the roles of pericytes in the pathogenesis of cerebrovascular diseases, including hemorrhagic brain injury, WMI, hypoxic–ischemic brain damage and other neurovascular dysfunctions, and clarified the progress in the treatment of cerebrovascular diseases targeted to pericytes.

## Biological Characteristics of Pericytes

### Research History of Pericytes

**FIGURE 1:**
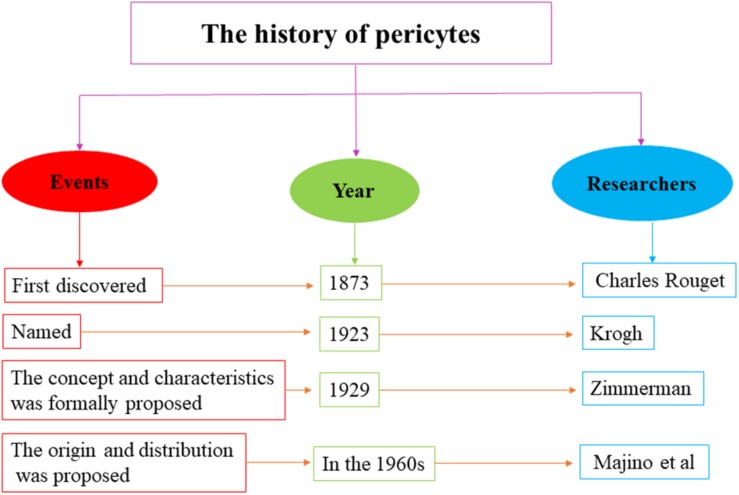
The research history of pericytes. The research history of pericytes covers several centuries, which ranges from first discovery in 1873 to further study in the 1960s.

The research history of pericytes covers several centuries. In 1873, pericytes were first discovered by Charles Rouget, who was investigating on capillary contractility. Later in 1923, Krogh named them “Rouget cells.” In 1929, the concept of pericytes was formally proposed by Zimmerman, who described their relevant biological characteristics. In the 1960s, Majino et al. showed that pericytes are derived from the mesenchyme of the mesoderm and are widely distributed in the capillaries of vertebrates (Figure 1; Dore-Duffy and Cleary, 2011). Subsequently, accumulating studies had investigated the source and destination of pericytes. Majino et al. indicated that CNS pericytes are mainly derived from the mesenchymal stem cells of the mesoderm and the neural crest cells of neuroepithelial origin (Faal et al., 2019). Cell transplantation experiments on chicken embryos have revealed that the pericytes of the forebrain are mainly differentiated from neuroepithelial cells (Etchevers et al., 2001), whereas the pericytes of the midbrain, brainstem, and spinal cord are derived from the mesoderm (Kumar et al., 2017). Moreover, human brain pericytes also contain a large amount of acid phosphatase and have a phagocytic function, suggesting that pericytes could be originated from macrophages (Barreto et al., 2019). Further studies have shown that pericytes have rich destinations, which can differentiate into fat, muscle, cartilage, and bone cells as well as other types, indicating that pericytes have stem cell characteristics (Ahmed and El-Badri, 2018). Collectively, overall findings indicate that pericytes can be derived from various origins and are capable of producing diverse cells.

### Morphology, Distribution, and Communication of Pericytes

Pericytes are polymorphic and usually appear as star-shaped or flat cells. The cell body of pericytes is spherical or oval and contains a large, round nucleus. The cytoplasm of pericytes contains a large number of organelles and mitochondria that can synthesize proteins, in addition to several lysosomes, which make the outer surface of pericytes appear to be granular (Jindatip et al., 2012). Studies have revealed that pericytes are constricted cells that spaced apart on capillary walls and closely linked to capillary endothelial cells. Besides, pericytes are widely distributed in the large blood vessels of the entire body, which located between the intima and adventitia of the microvascular and capillary walls, and mainly cover the capillaries, arterioles, and venules (Mazurek et al., 2017). Although pericytes are distributed throughout all organs, the number of CNS pericytes, which are important cellular components of the NVU and the BBB, is higher than that of pericytes in other organs (Armulik et al., 2011). In capillaries, pericytes are located between the endothelial cells and basement membrane, together with the endothelial cells, pericytes form a barrier between the blood vessels and interstitial space, such as the BBB, retinal blood barrier, and the like (Hall et al., 2014). Pericytes extend long protrusions along the longitudinal axis of the blood vessels that branch into two or three longitudinal and annular protrusions surrounding the blood vessels. The longitudinal protrusions are distributed along the longitudinal axis of the blood vessels, whereas the annular protrusions are perpendicular to the longitudinal axis. The neurites extended by pericytes contain large amounts of tubulin, whereas those on the surface of cell membranes that contact the endothelial cells contain dense microfilament-rich bands (Sava et al., 2017). However, when observed under a transmission electron microscope, pericytes are almost invisible, and the pericytes in mature capillaries have a disc-shaped nucleus surrounded by a small amount of cytoplasm (Liu et al., 2016). Based on the distribution and morphological characteristics of pericytes, the transmission of information between pericytes, and other cells requires the formation of specific structures. For instance, the communication between pericytes and endothelial cells requires the formation of gap junctions, adhesion plaques, and structures such as peg–socket boxes (periosteal cells forming staples and wrapped by nail grooves formed by endothelial cells). The discontinuity of the basement membrane and the incomplete coverage of pericytes allow the endothelial cells to form a broad connection with the glial border membrane, which in turn interacts with astrocytes, and together they form the NVU (Hartmann et al., 2015). In addition, *in vitro* studies showed that pericytes could express occludin, claudin-12, and zona occludens-1 and -2 on the cell border, suggesting that tight junctions can be formed between pericytes (Shimizu et al., 2008).

### Identification of Pericytes

Pericytes express a series of protein molecules, including α-SMA (Alarcon-Martinez et al., 2018), nestin (Muñoz-Fernández et al., 2018), vimentin (Bandopadhyay et al., 2001), NG2 (Sato et al., 2016), aminopeptidases A and N (Winkler et al., 2018), intercellular adhesion molecule-1 (ICAM-1) (Fan et al., 2019), vascular cell adhesion molecule-1 (Winkler et al., 2018), platelet-derived growth factor alpha and beta receptors (PDGFR-α and -β) (Wilson et al., 2018), CD34, CD146 (MCAM), CD4, CD11b (Wang et al., 2019), regulator of G-protein signaling 5 (RGS5) (Berger et al., 2005), stem cell antigen-1 (Kunisaki, 2019), and major histocompatibility complex classes I and II (Rink et al., 2017), without the expression of von Willebrand factor (Yin et al., 2019), platelet endothelial cell adhesion molecule (Nikolajsen et al., 2016), and glial fibrillary acidic protein (Winkler et al., 2018). Brain pericytes also express ATP-sensitive potassium channel protein Kir6.1 (Table 1; Bondjers et al., 2006). Nonetheless, due to the heterogeneity of pericytes, the surface markers of pericytes may be different even in different areas of the same tissue and even in different stages of differentiation or different pathophysiological states of the same pericytes (Dias Moura Prazeres et al., 2017). In addition, the surface markers of pericytes may not be specific for pericytes. For example, PDGFR, the common marker for pericytes, can also be expressed in fibroblasts and smooth muscle cells (Muñoz-Fernández et al., 2018). Therefore, in view of the deficiency of specific immunological molecular markers for pericytes, current approaches to pericytes identification are mainly based on morphology and the combined application of a series of positive and negative immunological markers. For instance, to identify musculoskeletal pericytes of vascular origin, researchers use combined detection of markers for CD146, NG2, α-SMA, and PDGFR expression without CD31, CD34, CD45, and CD144 (Tonlorenzi et al., 2017).

**TABLE 1:** The markers of pericytes.

**Markers**	**References**
α-SMA	Alarcon-Martinez et al., 2018
Nestin	Muñoz-Fernández et al., 2018
Vimentin	Bandopadhyay et al., 2001
NG2	Sato et al., 2016
Aminopeptidase A and N	Winkler et al., 2018
ICAM-1	Fan et al., 2019
VCAM-1	Winkler et al., 2018
PDGF-α,β	Wilson et al., 2018
CD34/CD146/CD4/CD11b	Wang et al., 2019
RGS-5	Berger et al., 2005
Sca-1	Kunisaki, 2019
MHC-I/II	Rink et al., 2017
Kir6.1	Bondjers et al., 2006

*α-SMA, alpha smooth muscle actin; NG2, chondroitin sulfate proteoglycan; ICAM-1, intercellular adhesion molecule-1; VCAM-1, vascular cell adhesion molecule-1; PDGF-α, β (platelet-derived growth factor α, β receptors); RGS-5, regulators of G protein signaling 5; Sca-1, spinocerebellar ataxia 1; MHC-I/II, major histocompatibility Complex-I and II; Kir6.1, ATP-sensitive potassium channel protein.*

### Culture of Pericytes

The heterogeneity of pericytes makes their identification complicated and difficult. It is easy to mix other cells (e.g., adventitial cells) with pericytes after isolation. The traditional methods for pericytes isolation include sequential sieve technique and immunomagnetic bead sorting. With the sequential sieve technique, the brain tissue is first converted into a cell suspension and sequentially passes through a sieve with different pores to separate the pericytes. This method requires initial separation of the microvascular fragments, which is cumbersome. Moreover, the grinding time is difficult to control, which may result in physical damage to the microvessels. Additionally, the separated microvascular fragments are usually not conducive to progeny cells climbing out, and cell viability is weak (Boroujerdi et al., 2014). In contrast, immunomagnetic bead sorting uses antibodies for the markers of pericytes linking to the magnetic beads to separate pericytes from other cells. The commonly used markers of pericytes include NG2, α-SMA, desmin, and PDGFR-β, which vary depending on the species, tissues, and developmental stages of the origin. Similarly, this method consists of a complicated process and is costly. Moreover, the effect of separation and culture is not ideal (Primo and Arboleda-Velasquez, 2016).

Considering that traditional culture approaches have their advantages and disadvantages, recent investigations have improved the technique of separating the pulmonary microvascular pericytes through the use of mechanical shearing, enzymatic digestion, mesh filtration, and lysis by red blood cell lysate to obtain a purer microvascular fragment. Given that pericytes can secrete TGF-β to inhibit endothelial cell growth, researchers have acquired pulmonary microvascular progeny cells with a purity of 100% by extending the natural purification time. Compared with the two former methods, this method avoids damage to the microvascular fragments during grinding and has a simpler operation process, thereby reducing the procedural time and cost. Additionally, the progeny cells are easily derived from the microvascular fragments, and cell survival probability is increased. Moreover, this approach solves endothelial cell contamination, which is the main problem when isolating pericytes by using microvascular fragments (Wertheim et al., 2019). Currently, isolation and *in vitro* culture technologies for the retinal, spinal, lung, and brain microvascular pericytes are relatively mature.

## Function of Pericytes

As one of the most important components of the blood vessels, pericytes carry out various physiological functions. The functions of pericytes can be roughly summarized as follows.

### Regulation of Vascular Genesis and Microecology

**FIGURE 2:**
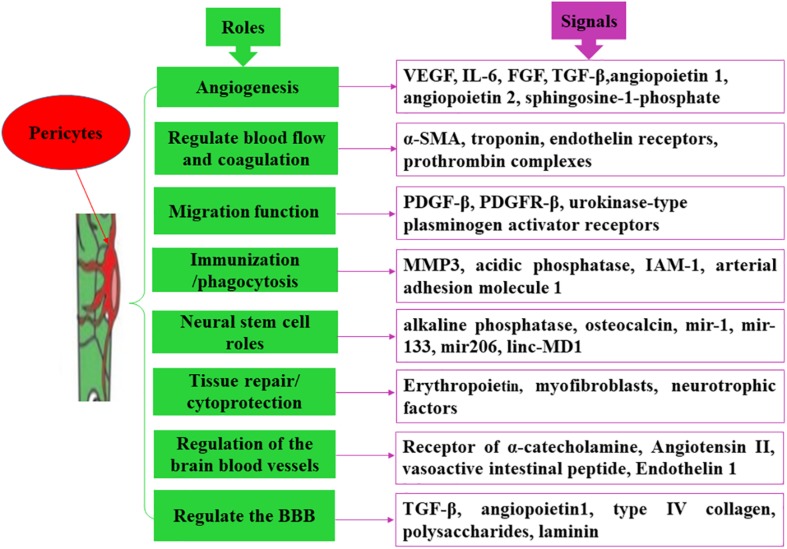
Main functions of pericytes. Pericytes exert divers functions via secreting different molecules.

Studies have shown that the formation of new blood vessels and the maintenance of vessel wall stability require a sufficient number of pericytes (Bergers and Song, 2005). Pericytes are the dominant cells in regulating angiogenesis, primarily via secretion of different signals. The process of angiogenesis involves four major steps (Diaz-Flores et al., 2017). In the initial stage of angiogenesis, pericytes promote endothelial cell maturation and neovascular sprouting by secreting vascular endothelial growth factor and interleukin-6 (IL-6) (Eilken et al., 2017). During the stage of shaping and prolongation of blood vessels, pericytes contribute to the migration, proliferation, aggregation, and differentiation of endothelial cells by secreting vascular endothelial growth factor and fibroblast growth factor (Cross and Claesson-Welsh, 2001). In the process of generation, connection, and termination of new blood vessels, an active interaction exists between pericytes, and endothelial cells. For instance, PDGFR-β secreted by endothelial cells should firstly bind to endothelium-derived heparan sulfate proteoglycan, which promotes pericytes recruitment around the blood vessels and facilitates peripheral cell proliferation and migration by interacting with the PDGFR-β receptor on the surface of pericytes (Cuervo et al., 2017). In the last stage of angiogenesis, pericytes divide and proliferate rapidly to generate the extracellular matrix, accelerate the maturation of neovascularization, and participate in the modification and reinforcement of new blood vessels (Figure 2; Marchand et al., 2018).

Pericytes signals are also involved in the construction of vascular microecology after ischemia. For example, in brain tissues, brain pericytes are capable of secreting angiopoietin-1, and stromal cell-derived factor-1. Angiopoietin-1 binds to Tie2 expressed by endothelial cells and promotes Tie2 auto-phosphorylation (activation), which in turn recruits peripheral cells, such as smooth muscle cells and pericytes, to support endothelial cells in forming intact blood vessel walls, promote vascular remodeling and maturation, maintain vascular integrity, and regulate vascular function (Fukuhara et al., 2010). At the same time, stromal cell-derived factor-1 and its specific receptor CXCR4 promote endothelial cell migration, adult blood vessel formation, nerve development, and neuronal migration (Figure 2; Cui et al., 2013).

### Regulation of Brain Blood Vessels

Pericytes have different subtypes and multiple functions. Pericytes located near the end of the capillary arteriole express more α-SMA and participate in the reflow process. These contractile pericytes contract the capillaries when ischemia occurs and are preferentially involved in the regulation of cerebral blood flow. Targeting these pericytes can reduce the no-reflux phenomenon after ischemic brain injury (Fernandez-Klett and Priller, 2015). The contraction of pericytes is involved in the following four mechanisms: (1) the endothelial cells surrounded by blood vessels show a flaky-fold structure with high and low undulations; (2) the ultrastructure of pericytes has a cytoskeletal structure capable of finely regulating cell contraction; (3) pericytes can express contractile proteins, such as α-SMA and troponin, and the expression levels of contractile proteins are distinct in different blood vessels; and (4) pericytes have some molecular receptors, such as endothelin receptors, which interact with angiotensin II and norepinephrine, causing rapid contraction of pericytes. Pericytes in the middle of capillaries express lower α-SMA and are preferentially involved in the maintenance of the BBB. Targeting these pericytes may reduce BBB damage after ischemic brain injury (Cai et al., 2017). However, the pericytes located at the end of the capillary venules are preferred to regulate the entry of immune cells into the brain parenchyma, and the amount of immune cells that enters into the brain parenchyma is significantly increased after ischemic brain injury (Navarro et al., 2016). Additionally, pericytes are capable of regulating blood pressure and coagulation. Angiotensin II secreted by endothelial cells can bind to its receptors on pericytes, causing contraction of the systemic arterioles, and elevation of arterial blood pressure, whereas vasoactive intestinal peptide secreted by endothelial cells can bind to its receptors on pericytes and has a vasodilating effect in lowering blood pressure (Fransen et al., 2016). Endothelin-1 is an endogenous vasoconstrictor regulator that is also present on pericytes (Dore-Duffy et al., 2011). During vascular injury or reconstruction, pericytes can secrete tissue factors, activate other coagulation factors, and form a positive feedback for the exogenous coagulation pathway, thereby regulating the prothrombin complex and participating in the whole coagulation process. At the same time, pericytes can also provide the necessary membrane surface for prothrombin complexes and promote the formation of thrombin with the help of coagulation factors Va and Xa. For example, human choroidal pericytes not only produce and regulate procoagulant complex but also support the functionally activated tissue factors present on its surface, which form a functional complex by binding to factor VIIa, thereby activating factors IX and X (Figure 2; Luo et al., 2018).

Collectively, pericytes regulate blood vessels by contracting themselves, controlling the size of capillary lumens, preventing excessive expansion of blood vessels, and maintaining homeostasis and metabolic balance of tissues.

### Regulation of BBB

Pericytes are one of the constituent cells of the NVU and play an important role in regulating the permeability of the BBB. Hypoxia can lead to increased permeability of monolayer endothelial cells, and co-culture with pericytes can reduce sensitivity to hypoxia and permeability of endothelial cells (Yamamizu et al., 2017). In addition, pericytes and brain microvascular endothelial cells communicate through gap junctions, tight junctions, focal adhesions, and some soluble factors, which in turn secrete growth factors to regulate the formation of tight junctions and permeability of endothelial cells that are important for the remodeling and maintenance of the vascular system (Ma et al., 2018). At the same time, pericytes can also regulate the proliferation, migration, and differentiation of endothelial cells (Zhou et al., 2016). Moreover, type IV collagen, polysaccharides, and laminin synthesized by the mouse kidney pericytes have been reported to have an effect on the formation of basement membrane (Wnuk et al., 2017). Hence, it can be speculated that pericytes are involved in the formation and maintenance of the BBB (Figure 2).

### Migration Function

During arterial vascular development, endothelial-derived PDGF-β and PDGF-β receptors expressed by pericytes mediate pericytes migration to the endothelial cells (Thijssen et al., 2018). *In vitro* experiments that mimic capillary structures of the human BBB have shown the migration of pericytes to endothelial cells (Campisi et al., 2018). In addition, cortical pericytes can rapidly respond under brain hypoxic conditions or during brain trauma, wherein approximately 40% of pericytes migrate to the perivascular site, which may be mediated by urokinase-type plasminogen activator receptors expressed by pericytes, and exacerbate BBB damage and increase BBB permeability, leading to cerebral edema (Figure 2; Castejon, 2011).

### Immunization and Phagocytosis

Pericytes have been reported to express matrix metalloproteinase 3 (MMP3); mice with MMP3 mutations exhibit a lower inflammatory response and a decrease in the number of permeable neutrophils, suggesting that pericytes are immunologically active in the inflammatory response (Kennedy-Lydon, 2017). At the same time, pericytes are capable of delivering antigens to T lymphocytes and participate in the immune response (Pober et al., 2017). Studies in the developing brain have revealed that lysosomes in pericytes can extensively express acidic phosphatase and exhibit phagocytic activity and that some pericytes can be directly transformed from macrophages (Prazeres et al., 2018). Furthermore, *in vitro* study found that cultured brain capillary pericytes can express a small number of adhesion molecules (such as ICAM-1 and arterial adhesion molecule-1) under the induction of inflammatory cytokines and can actively absorb and eliminate harmful small molecule particles (Pieper et al., 2014). These results indicated that pericytes have a phagocytic function that is similar to that of macrophages (Figure 2).

### Potentiality of Neural Stem Cell

Pericytes isolated from different tissues can differentiate into other types of cells. For example, when cultured *in vitro*, the expression levels of alkaline phosphatase and osteocalcin in pericytes are increased, aggregated, and calcified, and pericytes demonstrate certain osteogenic ability (Gaceb et al., 2018). Pericytes can also differentiate into odontoblasts (Farahani et al., 2012). Furthermore, when pericytes extracted from skeletal muscle vessels are implanted into rats with muscle dysfunction, they can differentiate into skeletal muscle cells, thereby producing a large number of muscle tubules, and inducing muscle remodeling (Pierantozzi et al., 2016). When pericytes extracted from bovine renal microvessels are transplanted into the rats, they can differentiate into cartilages and adipocytes (Konig et al., 2016; Vidhya et al., 2018). In addition, in hemorrhagic cerebral infarction, brain pericytes can differentiate into glial cells (Cheng et al., 2018). For different cell-derived and subtype pericytes, their differentiation and repair ability are not alike. For example, skeletal muscle-derived pericytes express three major myogenic RNA (mir-1, mir-133, and mir206) and unencoded myogenic RNA (linc-MD1), and their myogenic capacity was significantly higher than that of adipose-derived pericytes (Figure 2; Gautam and Yao, 2019).

### Tissue Repair and Cytoprotection

Pericytes are involved in the repair and regeneration of various tissues and organs. Under physiological conditions, renal pericytes can produce erythropoietin to maintain microvascular stability. However, during renal injury, pericytes are exfoliated from the blood vessels, migrating, aggregating, and proliferating toward the injury, where they differentiate into other types of cells (such as myofibroblasts) to promote capillary regeneration at the site of injury and prevent the loss of capillaries (Shaw et al., 2018). Additionally, brain pericytes can express neurotrophic factors to promote neuronal survival when hypoxia occurs (Li et al., 2017). In the cardiovascular system, pericytes are involved in angiogenesis and affect the stability of arterial plaques. For instance, arterial plaques contain a large number of new blood vessels; when pericytes are absent, the permeability and fragility of new blood vessels increase, leading to plaque instability (Kennedy-Lydon, 2017). Pericytes also control effective myocardial perfusion by regulating the contractility of myocardial capillaries and participate in the reconstruction process after myocardial infarction (Costa et al., 2018). However, during the process of tissue repair, pericytes can cause excessive fibrosis. When repairing renal damage, pericytes may reduce the stability of the glomerular microvasculature, resulting in chronic hypoxia in the tubular and interstitial cells, and in turn leading to renal fibrosis (Venkatachalam and Weinberg, 2017). Pericytes can also cause pulmonary fibrosis and hepatic fibrosis. Along with increasing age, the activity of pericytes decreases and some special receptors are also lost, hindering the related signaling pathways for differentiation into other tissues, and thereby reducing the regeneration function for tissue repair (Figure 2; Birbrair et al., 2015).

## Pericytes and Cerebrovascular Diseases

The neurovascular unit is a dynamic functional module composed of various members, such as neurons, glial cells, and pericytes. Studies have shown that NVU dysfunction is involved in the development of various cerebrovascular diseases, including hypoxic–ischemic brain damaged, brain WMI, and cerebral hemorrhage (Hill et al., 2014). Pericytes can regulate nerve and blood vessel events. For example, brain pericytes are involved in the regulation of cerebral blood flow, neurovascular growth, homeostasis of the NVU, and development and integrity of the BBB, and they have the potential activity of immune phagocytosis and stem cells (Sa-Pereira et al., 2012). In addition, studies on PDGFR-β-deficient rats reported that loss of brain pericytes might cause nerve damage through a reduction in chronic hypoperfusion and hypoxia by decreasing the level of brain microcirculation and through massive accumulation of plasma proteins and neurotoxic substances owing to damage to BBB integrity (Sauer et al., 2017). Both processes are related to the activity of pericytes.

### Pericytes and Cerebral Hemorrhage

After cerebral hemorrhage, thrombin induces the release of MMP9, and its expression in brain pericytes is higher than that in other BBB-constituting cells, which may be related to the higher expression of thrombin receptors PAR1 and PAR4 in brain pericytes (Underly et al., 2017). Among proteins, PAR1 is the most abundant in brain pericytes, and the use of PAR1 inhibitor SCH79797 can prevent the release of MMP9 from brain pericytes, suggesting the important role of the thrombin–PAR1–MMP9 axis of brain pericytes in the pathological process of cerebral hemorrhage (Machida et al., 2017). Further studies have shown that thrombin stimulates PAR1 in brain pericytes after cerebral hemorrhage, activates PKCh-Akt and PKCd-ERK1/2 signaling pathways, and subsequently releases MMP9, leading to BBB damage, and aggravating brain damage (Table 2; Wang et al., 2018).

**TABLE 2:** The pericytes with cerebrovascular disease.

**Cerebrovascular**	**Pericytes signals/**	**References**
**disease**	**function**	
Cerebral hemorrhage	PAR1 PAR4 MMP9	Machida et al., 2017; Underly et al., 2017; Wang et al., 2018
Hypoxia-ischemic brain damage	Calcium overload Pericyte excessive contraction TGF-β1/AP-1 Collagen IV/laminin/hyaluronic acid	Kose et al., 2007; Zechariah et al., 2013; Rustenhoven et al., 2016; Cai et al., 2017
White matter injury	Notch 3 Kir6.1/ATP	Thomzig et al., 2005; Kofler et al., 2015
Neovascular deficiency disease	PHD2 VEGF PDGFβ RGS5 SPARC Sema3A/Nrp-1	Hamzah et al., 2008; Dhar et al., 2010; Bai et al., 2015; Svensson et al., 2015; Urrutia et al., 2016; Thijssen et al., 2018

*PAR1, protease-activated receptor-1; PAR4, protease-activated receptor-4; MMP9, matrix metallopeptidase 9; AP-1, activator protein-1; PHD2, proline hydroxylase 2; VEGF, vascular endothelial growth factor; PDGFβ, platelet-derived growth factor β; RGS5, regulators of G protein signaling 5; SPARC, protein acidic and rich in cysteine; Sema3A, axon guiding molecule 3; Nrp-1, neurociliary protein 1.*

### Pericytes and Hypoxic–Ischemic Brain Damage

Cerebral hypoxic–ischemic damage triggers the activity of pericytes. However, whether the activation of pericytes is beneficial or harmful depends on the specific tissue microenvironment, degree of activation, type of stimulation, and existing factors (Schneider et al., 2015). For instance, pericytes take on various tasks in different stages of ischemic stroke (Cai et al., 2017). (1) In the hyper-acute phase of stroke, the intracellular calcium concentration is enhanced, the number of ATP molecules is insufficient, and oxygen and nitrogen free radicals are formed in the microvasculature, leading to the contraction and death of pericytes, which finally cause no-reflow phenomenon in the capillaries of the brain. (2) In the acute phase, pericytes are stripped from microvessels and participate in inflammatory and immune responses, leading to BBB damage and cerebral edema. (3) During the late recovery phase of stroke, pericytes contribute to angiogenesis and neurogenesis, thereby promoting nerve recovery. In addition, pericytes are abnormally active and exhibit quantitative, structural, and functional changes when stimulated by certain extracellular noxious stimuli (such as acidosis, high glucose level, and reactive oxygen species), which cause pericytes dysfunction and excessive contraction, hinder red blood cells from passing through the vessels, and subsequently lead to microcirculatory disorders. For example, in a rat model of cerebral ischemia, oxidation-nitrous stress can induce constant contraction of retinopathy pericytes, and result in the disturbance of microcirculation and blood flow (Fu et al., 2016). In focal ischemic rat models, the contraction of pericytes occurred during cerebral artery occlusion and persisted even after the blood flow was restored (Zechariah et al., 2013). Thus, excessive and continuous contraction of pericytes after cerebral ischemia may be the cause of local microcirculation disorder, which may also be an important pathological mechanism of cerebral ischemia–reperfusion injury (Table 2).

Nevertheless, pericytes also provide benefits for neuroprotection in hypoxic–ischemic brain damage by protecting the endothelium, stabilizing BBB permeability, and releasing neurotrophins (Ishitsuka et al., 2012). For example, in the *in vitro* BBB model with mixed endothelial cells, pericytes, and astrocytes, the researchers found that the permeability of monolayer endothelial cells was significantly attenuated due to the presence of pericytes. Similarly, when co-cultured with pericytes, the sensitivity of endothelial cells to hypoxia was significantly reduced (Wang et al., 2016). Under continuous hypoxic conditions, TGF-β1 and angiopoietin-1 expressed by pericytes contributed to the maintenance of the tight junction of the BBB (Rustenhoven et al., 2016), whereas type IV collagen, laminin, and hyaluronic acid secreted by pericytes are involved in the construction of the basement membrane (Kose et al., 2007). In addition, when observing the brain tissue of rats with minimal cerebral hemorrhage via electron microscopy, researchers found that ions, and plasma components exist in the cytoplasm of pericytes (Jindatip et al., 2018). Therefore, when the tight binding of the BBB was disrupted, pericytes might act as phagocytes to play a remedial role (Table 2).

### Pericytes and Brain White Matter Injury

Brain WMI can be induced by hypoxic–ischemia, infection, and so on (Duerden et al., 2019). Pericytes dysfunction is involved in the pathogenesis of WMI (Montagne et al., 2018). Numerous studies have confirmed that human *Notch3* gene is mainly expressed in the arterial smooth muscle cells and pericytes and that its mutations cause capillary blood flow dysregulation, thereby triggering impaired cerebrovascular reactivity and WMI disorders such as hereditary multi-infarct dementia (cerebral autosomal dominant arteriopathy with subcortical infarcts and leukoencephalopathy) (Kofler et al., 2015). Furthermore, the observation of cerebral microvessels in patients with cerebral autosomal dominant arteriopathy with subcortical infarcts and leukoencephalopathy via electron microscopy indicated that pericytes showed degeneration, the nuclei were swollen, and the basement membrane between pericytes and endothelial cells was thickened with a large amount of osmiophilic granules (Craggs et al., 2015). In rats with chronic cerebral hypoperfusion-induced WMI, the pericytes were damaged and its biomarker Kir6.1/Kir6.2 was expressed in heterogeneity, suggesting that the Kir6.1/K-ATP channel might be involved in the pathological process of chronic WMI (Table 2; Thomzig et al., 2005).

### Pericytes and Neovascular Deficiency Disease

Pericytes maintain vascular osmotic pressure and morphology and participate in vascular genesis and maturation via signal transmissions with endothelial cells and extracellular matrix (Ferland-McCollough et al., 2017). The insufficiency of pericytes always leads to the occurrence and development of neovascular deficiency disease (Bergers and Song, 2005). The neovascular deficiency disease refers to a category of disorder which is caused by the shortage of formation or immaturities of new blood vessels, including tumors, atherosclerosis, diabetic retinopathy, and the like. Besides, neovascular deficiency often occurs following different types of brain damage such as hypoxic ischemic encephalopathy and WMI, which in turn attenuated the recovery of brain injury. Generally, this category of disorder shares the following characteristics: (1) the morphology of endothelial cells is abnormal, showing overlapping growth, and the connection between endothelial cells is reduced; (2) the morphology of pericytes is abnormal, the function is inadequate, and the coverage is decreased; and (3) the basement membrane is incomplete or missing, with uneven thickness or thinness, and is distributed in sheets (Davis et al., 2015). Studies have shown that these neovascular immaturity diseases are associated with various signaling pathways. For example, the excessive activation of angiogenic signaling pathways, such as proline hydroxylase-2 (Urrutia et al., 2016) and vascular endothelial growth factor (Bai et al., 2015), leads to excessive angiogenesis. On the other hand, the recruitment and proliferation of pericytes are inhibited and the decomposition of the basement membrane is excessive, ultimately inhibiting the maturation of new blood vessels (Cheng et al., 2018). In addition, the signals secreted by pericytes such as PDGF-β (Thijssen et al., 2018), RGS5 (Hamzah et al., 2008), secreted protein acidic and rich in cysteine (Svensson et al., 2015), and axon guidance molecule 3/neurociliary protein 1 (Sema3A/Nrp-1) (Dhar et al., 2010) are also closely related to the occurrence of neovascularization immaturity (Table 2).

## Pericytes and Non-Vascular Brain Disorders

### Pericytes and Depression

Depression is a type of neuropsychiatric disease caused by NVU dysfunction, which is closely related to neuroinflammation that can affect serotonin levels (Liu et al., 2015). Depression is also associated with NVU dysfunction caused by abnormally high BBB permeability. For example, studies have shown that the BBB integrity in patients with depression is disrupted, and the pericytes coverage of the cortical and hippocampal capillary networks is significantly reduced (Liu et al., 2019). In addition, *in vitro* study indicated that brain pericytes secrete various cytokines, including ICAM-1, IL-6, tumor necrosis factor-α (TNF-α), and MMP, which disrupt BBB integrity and aggravate brain inflammatory factors (Dohgu et al., 2019). Numerous studies have shown that inflammatory factors such as IL-6, IL-1, and TNF-α can trigger acute pro-inflammatory effects and that the downregulation of these factors can induce depression-like behavior (Chu et al., 2019). However, the upregulation of IL-6, IL-1β, and TNF-α has been shown to impair nerve cell regeneration and aggravate cell death, leading to neurodegenerative diseases (Zou et al., 2018). In addition, neuropathological studies have reported that patients with depression have abnormally elevated ICAM-1 in the prefrontal cortex and white matter. Antidepressant treatment reduces the expression of vascular cell adhesion molecule-1 and ICAM-1 in serum. These effects are triggered primarily by the interference of these cytokines secreted by pericytes, which disrupt cell survival signaling pathways and caspase-dependent cascades, and altering the function of major receptors (Dewispelaere et al., 2015).

### Pericytes and Alzheimer’s Disease

Alzheimer’s disease, triggered by chronic inflammation as well as the pathological accumulation of beta-amyloid (Aβ) and neurofibrillary tangles in the brain, is related to pericytes dysfunction (Yamazaki and Kanekiyo, 2017). In transgenic animals with AD pathology, researchers found that circulating white blood cells penetrate the BBB to interact with NVU components and disrupt their structural integrity and function (Zuo et al., 2017). In addition, Aβ receptors such as LRP1, LDLR, RAGE, and CD36 are expressed on pericytes in the brain of AD patients, and these receptors are involved in Aβ-mediated cerebral perivascular cell death (Miners et al., 2018). Mice with depleted pericytes showed deposition of Aβ plaques in the brain parenchyma and blood vessels, as well as an accumulation of hyper phosphorylated tau protein, suggesting that pericytes deficiency contributed to the development of tau pathology (Sagare et al., 2013).

## Targeting Pericytes as a Therapeutic Strategy for Cerebrovascular Diseases

Pericytes play an important role in the maintenance of normal functions of blood vessels in the brain and are involved in the pathogenesis of several cerebrovascular diseases. Therefore, targeting pericytes is a potential therapeutic strategy for cerebrovascular diseases.

Regulator of G-protein signaling 5, which regulates vascular development, showed a perivascular expression pattern and was identified as a marker for brain pericytes (Bondjers et al., 2003). In a mouse model of permanent midbrain occlusion, when knocking out *RGS5* gene, the amount of pericytes and endothelial coverage were increased, and the BBB injury was also significantly attenuated. Furthermore, loss of *RGS5* in pericytes alleviated vascular leakage, maintained tight junction integrity and aquaporin-4 levels, reduced cerebral hypoxia, and protected neurons in the infarcted area (Ozen et al., 2018). Therefore, targeting pericytes RGS5 might be effective in the treatment of ischemic stroke.

In a mouse model of cerebral ischemia, when TGF-β was used, it continuously stimulated the formation of extracellular matrix and the transformation of mesenchymal cells to vascular pericytes, thereby strengthening the blood vessels, preventing the leakage of new blood vessels, and promoting the maturation of blood vessels (Dhandapani and Brann, 2003). Therefore, TGF-β might be an effective agent to treat neovascular deficiency disease.

In a rat model of cerebral ischemia–reperfusion injury, pericytes were observed to migrate from the vessel wall at an early stage, the microvascular basement membrane structure was disordered, and the BBB function was impaired. When ischemic post-conditioning was performed, the MMP9 expression of pericytes was reduced. As MMP9 is a critical factor regulating pericytes migration, the downregulation of MMP9 after post-conditioning inhibited pericytes migration after ischemia–reperfusion, and maintained the integrity of BBB structure and function (Underly et al., 2017).

## Discussion

As components of the NVU, pericytes play an important role in the regulation of physiological and pathological processes of the CNS and have currently attracted considerable interest (Geranmayeh et al., 2019). This review primarily discussed the basic characteristics and research dynamics of pericytes in cerebrovascular diseases, aiming to provide a comprehensive understanding of pericytes and new insights about the use of pericytes as novel therapeutic targets for cerebrovascular diseases.

## Author Contributions

XS and YQ contributed to the conception and design of the review and drafting the manuscript. LH and DX participated in the table making work. YQ and DM contributed to approving the final version of the manuscript submitted for publication.

## Conflict of Interest

The authors declare that the research was conducted in the absence of any commercial or financial relationships that could be construed as a potential conflict of interest.

## Abbreviations

α-SMA: alpha smooth muscle actin
AD: Alzheimer’s disease
BBB: blood–brain barrier
CNS: central nervous system
ICAM-1: intercellular adhesion molecule 1
IL-6: interleukin 6
MMP3: matrix metallopeptidase 3
MMP9: matrix metallopeptidase 9
NG2: chondroitin sulfate proteoglycan
NVU: neurovascular unit
PAR1: protease-activated receptor-1
PAR4: protease-activated receptor-4
PDGFR- α β: platelet-derived growth factor α, β receptors
RGS5: regulators of G protein signaling 5
TGF- β: transforming growth factor
TNF- α: tumor necrosis factor α
WMI: brain white matter injury.
